# Anxiety and Attentional Bias to Threat in Children at Increased Familial Risk for Autism Spectrum Disorder

**DOI:** 10.1007/s10803-016-3012-1

**Published:** 2017-01-23

**Authors:** Bosiljka Milosavljevic, Elizabeth Shephard, Francesca G. Happé, Mark H. Johnson, Tony Charman, The BASIS Team

**Affiliations:** 1 Department of Psychology, Institute of Psychiatry, Psychology and Neuroscience, King’s College London, De Crespigny Park, London, SE5 8AF UK; 2 MRC Social, Genetic & Developmental Psychiatry Centre, Institute of Psychiatry, Psychology and Neuroscience, King’s College London, De Crespigny Park, London, SE5 8AF UK; 3 Centre for Brain and Cognitive Development, Birkbeck College, University of London, Malet Street, London, WC1E 7HX UK

**Keywords:** Autism Spectrum Disorder, High-risk siblings, Anxiety, Threat bias

## Abstract

Anxiety and threat bias were examined in 6-8-year-old children at familial-risk for Autism Spectrum Disorder (ASD) and low-risk (LR, *n* = 37) controls. The high-risk (HR) group was divided into those who met diagnostic criteria for ASD (HR-ASD, *n* = 15) and those who did not (HR-non ASD, *n* = 24). The HR-ASD group had highest levels of parent-reported anxiety. The HR-non ASD group exhibited increased threat bias on a spatial-cueing task, while the HR-ASD group did not. Anxiety symptoms were associated with both threat bias and ASD severity. These findings suggest that the mechanisms underlying anxiety in HR siblings without ASD are similar to those in non-ASD populations. However, among children with ASD, hypersensitivity to threat may not underlie anxiety symptoms.

**Electronic supplementary material:**

The online version of this article (doi:10.1007/s10803-016-3012-1) contains supplementary material, which is available to authorized users.

## Introduction

Individuals with Autism Spectrum Disorder (ASD) exhibit difficulties in social communication and relating, as well as restricted and repetitive patterns of behaviour and atypicalities in sensory modulation (DSM-5; American Psychological Association 2013). There is a 1% prevalence rate of ASD in the general population (Baird et al. 2006; Christensen et al. 2016), while recurrence rates in younger siblings of children with ASD are ~10% (Constantino et al. 2010). Prospective high-risk studies have reported that up to 20% of siblings actually meet diagnostic criteria for ASD and that there is increased ASD symptomatology among those that do not have the condition (Messinger et al. 2013, 2015; Ozonoff et al. 2011). Taken together, these findings suggest that there is high familial risk for ASD.

In addition to the core symptoms, individuals with ASD frequently experience co-occurring mental health difficulties, notably anxiety disorders (Simonoff et al. 2008). Up to 80% of individuals with ASD report anxiety symptoms, which are often impairing (White et al. 2009). Increased prevalence of anxiety is also reported in first-degree relatives of individuals with ASD (Lainhart 2009), including young children at increased familial risk as siblings of probands with a diagnosis (Schwichtenberg et al. 2013). Hallett et al. (2013b) directly compared anxiety symptoms in probands with ASD and their twins, who were either typically developing (TD) or manifested aspects of the Broader Autism Phenotype (BAP), sub-clinical traits of autism in family members (Bolton et al. 1994). The findings suggested that anxiety was most highly elevated among the ASD probands and twins with BAP.

While elevated rates of anxiety have been observed in individuals with ASD and their siblings, there is a scarcity of research examining the shared underlying neurocognitive mechanisms of the two conditions. Wood and Gadow (2010) suggest that such investigation is highly relevant, as it is presently unclear whether the co-occurrence of ASD and anxiety represents a true comorbidity, the manifestation of two separate conditions in the same individual, or if it results from an overlap in symptom presentation and difficulties with self- and caregiver-report. One way to better understand the manifestation of anxiety within ASD is to examine whether the neurocognitive mechanisms that are associated with anxiety in non-ASD populations, such as increased attentional allocation to threat, are also present and relate to anxiety symptoms in children with ASD and their siblings.

The present study addresses the association between anxiety symptoms and attentional allocation to threat within the context of a prospective longitudinal study of children at high familial risk for ASD (due to having an older sibling with the condition). This design provides a unique opportunity to examine the cognitive correlates of anxiety in high-risk siblings who themselves meet diagnostic criteria for ASD and those who do not. This allows for the comparison of siblings with ASD and those who go on to have typical development, as well as the examination of the relationship between anxiety, attention to threat and sub-clinical traits of ASD.

### Attentional Bias to Threat and Anxiety

Cognitive theories of anxiety disorders posit that highly anxious individuals may be particularly sensitive to threat-relevant information in the environment (Bar-Haim et al. 2007). Biased processing of threat is thought to contribute to both the development and maintenance of anxiety disorders (Beck and Clark 1997; Eysenck 1992). This cognitive style has been demonstrated experimentally using a number of tasks that compare reaction times (RTs) to threatening and non-threatening stimuli (for review see Bar-Haim et al. 2007). Studies using the dot-probe paradigm, one of the most widely used measures of attentional bias (Macleod et al. 1986), report that individuals with heightened anxiety are faster to detect a probe that has previously been paired with a threatening (compared to a neutral) stimulus, suggesting that they are hypervigilant for threat-relevant information (Macleod et al. 1986; Mogg and Bradley 1999).

However, the dot-probe paradigm has received criticism for not differentiating between different components of attention. Fox et al. (2001) argue that faster RTs to threatening stimuli may be a consequence of delayed disengagement from, rather than faster orienting to, threatening stimuli. Studies using paradigms that disentangle different facets of attention corroborate the postulation that anxiety is specifically associated with delayed disengagement from threatening stimuli, but not faster orienting towards it (Yiend and Mathews 2001; Salemink et al. 2007). This may be particularly relevant for individuals with ASD, who exhibit difficulties in flexibly shifting attention (Elsabbagh et al. 2013; Landry and Bryson 2004). Perhaps this cognitive style also contributes to cognitive processing in anxiety among individuals with ASD, resulting in more difficulty in shifting attention away from threat.

Given that most anxiety disorders first manifest in childhood (Beesdo et al. 2009), assessing threat bias among school-aged children at-risk for ASD may be particularly relevant in describing the early processes associated with the development of anxiety in this population. The association between threat bias and anxiety has been reported in both adults and children, but Dudeney et al. (2015) suggest that the strength of this association increases with age from early childhood to adolescence. Nevertheless, several studies using RT paradigms have reported that children as young as preschool-age with heightened anxiety exhibit both faster detection of and slower disengagement from threatening stimuli (Mian et al. 2015; Briggs-Gowan et al. 2015; Bar-Haim et al. 2011).

While threat bias has been studied very extensively among individuals with anxiety disorders, there is a dearth in research investigating this among ASD populations and studies to date have yielded equivocal results. Two studies examined attentional bias to angry faces and found that young people with ASD and elevated anxiety did not exhibit enhanced engagement to or delayed disengagement from threat, compared to participants with ASD who did not have heightened anxiety or TD controls (Hollocks et al. 2013; May et al. 2015). On the other hand, using an eye-tracking paradigm, White et al. (2015) found that prolonged fixation to threatening faces, depicting expressions of disgust and anger, was associated with fear of negative social evaluation (a construct linked to social phobia) in adolescents with ASD. In contrast to these studies, Isomura et al. (2015) found that children with ASD, who did not have clinical-level anxiety symptoms, exhibited prolonged disengagement from threatening (snakes) compared with non-threatening (flowers) stimuli. While it is not unusual to find a general bias to threat in children and adults (Lobue and Deloache 2008), participants with ASD had longer disengagement from the threatening stimuli than TD controls. It is important to note that, although participants in this study did not have clinical diagnoses of anxiety, subclinical symptoms or traits were not measured. Given that delayed disengagement is frequently observed among individuals with ASD and anxiety symptoms were not measured, it is unclear whether the attentional bias to threat reported in this study is a consequence of ASD symptoms, anxiety, or an interplay of both.

### Social and Non-Social Threat Stimuli

One of the limitations of previous studies examining threat bias in ASD is the use of human facial expressions as stimuli. There is a broad literature suggesting atypical face processing and emotion recognition among individuals with ASD (e.g. Harms et al. 2010). A recent meta-analysis suggests that individuals with ASD exhibit reduced performance on tasks that measure emotion recognition, particularly for negative emotions such as anger and fear (Uljarević and Hamilton 2013). Multiple studies also report both reduced accuracy in emotion labelling and attenuated neural activity when viewing emotional faces among first-degree relatives of individuals with ASD (Sucksmith et al. 2013; Spencer et al. 2011; Oerlemans et al. 2014). In the context of this evidence, the use of threatening facial expressions as stimuli may not be salient enough to detect an association between anxiety and attentional bias among ASD populations. On the contrary, individuals with ASD have exhibited heightened neural responses to unpleasant non-social stimuli, comparable to neural activity observed in TD controls (Silani et al. 2008), which is perhaps why bias to images of snakes compared to flowers was observed in children with ASD (Isomura et al. 2015). An additional challenge exists in selecting appropriate non-threatening comparison stimuli. Children with ASD often exhibit fears and phobias of unusual or commonplace objects (Mayes et al. 2013; Kerns et al. 2014). As a consequence, the traditional use of neutral stimuli may not be as clearly non-threatening to children with ASD. Perhaps more clearly positively valenced stimuli may be more effective in detecting differences in attentional allocation to threatening and non-threatening information.

### The Present Study

The present study sought to extend current understanding of anxiety in ASD by examining the association between parent-reported anxiety and threat bias, in a cohort of children at high familial risk for ASD (HR), some of whom met diagnostic criteria for ASD (HR-ASD) and others who did not (HR-non ASD), compared to low-risk (LR) controls. Importantly, we aim to address limitations in previous work by examining bias to non-social threatening stimuli, which may be more salient among children with ASD. In our recent work with this cohort, we found that anxiety was substantially elevated in the HR children, most highly among those who were HR-ASD and to a slightly lesser degree among HR-non ASD children (Shephard et al. 2016). The present study focuses primarily on parent-reported anxiety both due to the young age of our participants and previous reports that children with ASD may have difficulty reflecting on their internal states and under-report symptoms of anxiety (e.g. Mazefsky et al. 2011).

Given the present literature, this study aims to address the following hypotheses:

1. Children at HR for ASD will show evidence of attentional threat bias. In light of the literature suggesting that anxiety may be associated with prolonged disengagement from threat (Fox et al. 2001) and reports that children with ASD have difficulty in flexibly shifting attention (Elsabbagh et al. 2013; Landry and Bryson 2004), we predict that threat bias will be observed through delayed disengagement from, rather than faster orienting to, threatening stimuli.
2. Previous findings suggest that anxiety is highly elevated among siblings who themselves have ASD, and also (albeit to a lesser degree) among those who do not have ASD (Hallett et al. 2013b; Shephard et al. 2016). Therefore, we predict that threat bias will also be highest among children in the HR-ASD group, followed by those who are HR-non ASD, and lowest in LR controls.
3. Since children with ASD report heightened fear of atypical or commonplace objects (Mayes et al. 2013; Kerns et al. 2014), threat bias will be more readily observed when comparing threatening with positive, rather than threatening with neutral, stimuli within the HR sample.
4. Finally, there will be an association between anxiety symptom severity and attentional threat bias, regardless of ASD severity.

## Method

### Participants

Fifty-four children at high-risk (HR) and 50 children without a family history (LR) of ASD were recruited through the British Autism Study of Infant Siblings (BASIS; http://www.basisnetwork.org), a prospective longitudinal study of infants at increased familial risk for ASD. Research visits took place when the children were aged 7, 14, 24 and 36 months and 6–8 years (hereafter the ‘7-year visit’). HR infants (21 males, 33 females) were recruited on the basis of having an older sibling with a community clinical diagnosis of ASD. These diagnoses were confirmed by two expert clinicians (TC, PB) with information from the Development and Wellbeing Assessment (DAWBA; Goodman et al. 2000)^1^ and the Social Communication Questionnaire (SCQ; Rutter et al. 2003)^1^. Additionally, family medical history was collected through parent-report, to screen for related conditions (e.g. Fragile X syndrome, tuberous sclerosis) in the proband or extended family members but no such conditions were reported. LR controls (21 males, 29 females) were recruited from the Birkbeck Centre for Brain and Cognitive Development volunteer database. LR infants’ medical histories were screened and confirmed that the infants were born at full-term^2^ and that there was an absence of ASD in their first-degree relatives. All LR infants had at least one older sibling and absence of ASD in older siblings was confirmed using the SCQ, with no child scoring above cut-off (≥15)^3^.

Of the 104 children initially recruited, 44 HR and 37 LR took part in the 7-year follow-up. However, two children did not complete research visits (parents completed questionnaires only) and we were unable to assign them to an ASD outcome group and, as a result, excluded them from further analyses. Participants that were retained at the present visit did not differ on measures of ASD symptoms (ADOS, SRS, SCQ), adaptive functioning (Sparrow et al. 2005) or developmental level (Mullen 1995) from the non-retained participants (min. *p* = .40). The final sample consisted of 42 HR children (15 males, 27 females) and 37 LR controls (15 males, 22 females). At the 7-year visit, parent-report of participants’ medical and family histories were collected and revealed that 5 children in the HR group were from multiplex families with multiple siblings with a community clinical diagnosis of ASD, while the rest were from simplex families. Furthermore, none of the children had been diagnosed with relevant medical conditions (e.g. Tuberous Sclerosis, Fragile X). However, 4 HR children were reported to have experienced seizures in early childhood, although these had ceased in all cases. None of the children had a formal diagnosis of an anxiety disorder, although one child in the LR group was receiving treatment for anxiety-related issues. Participants were assigned to an ASD outcome group at the 7-year visit, based on measures of ASD symptoms (ADOS-2, ADI-R, SCQ) as described below.

Ethical approval was obtained from the NHS National Research Ethics Service (NHS RES London REC 14/LO/0170). Parents provided written informed consent. Children provided written informed assent wherever possible given developmental level.

### Measures of ASD Symptomatology

The Autism Diagnostic Observation Schedule—2nd Edition (ADOS-2; Lord et al. 2012), is a standardised, semi-structured observational assessment of ASD symptoms, focusing particularly on communication, social interaction, play and restricted and repetitive behaviours. Calibrated Severity Scores (CSS) for Social Affect (SA), Restricted and Repetitive Behaviours (RRB) and total score were computed and provide standardised ASD severity based on the module administered and the participant’s age and verbal ability (Gotham et al. 2009; Hus et al. 2014). Within our sample, Module 3 was used for 73 children, Module 2 for one child, Module 1 for one child, and 3 LR controls did not complete the assessment.

The Autism Diagnostic Interview-Revised (ADI-R; Le Couteur et al. 1989) is a standardised, semi-structured clinical interview that provides a diagnostic algorithm for autism in accordance with both ICD-10 and DSM-IV criteria. The assessment focuses on three domains: Reciprocal Social Interaction, Communication, Restricted, Repetitive and Stereotyped patterns of behaviour, as well as onset of symptoms. Within the present sample, the ADI-R was administered only to children in the HR group.

The Social Communication Questionnaire (SCQ; Rutter et al. 2003) lifetime version was completed by parents and was used to evaluate communication skills and social functioning related to ASD. The questionnaire contains 40 items and a score ≥ 15 indicates the presence of ASD.

The Social Responsiveness Scale-Second Edition (SRS-2; Constantino 2012) was completed by parents and was used to evaluate the severity of social difficulties associated with ASD. The questionnaire consists of 65 items, which provide a total score of autistic traits.

### Cognitive Functioning

The Wechsler Abbreviated Scales of Intelligence-Second Edition (WASI-II; Wechsler 2011) was used to measure general cognitive ability and provides standardised, age-normed intelligence quotients for verbal comprehension (VCI), perceptual reasoning (PRI), and full-scale IQ (FSIQ). We included measures of IQ due to vast evidence suggesting that cognitive ability is related to performance on experimental tasks that measure RT (for review see Sheppard and Vernon 2008).

### Assignment to ASD Outcome Group

Assignment to ASD outcome group was conducted at the 7-year visit. The clinical measures of ASD symptomatology (ADOS-2, ADI-R, SCQ), as well as information from all previous visits, were reviewed by four experienced researchers (ES, BM, GP, TC) to establish an ASD diagnostic outcome according to DSM-5 criteria (American Psychological Association 2013). Subsequently, children in the HR group were divided into those who met diagnostic criteria for ASD (HR-ASD, *n* = 15) and those who did not (HR-non ASD, *n* = 27). None of the 37 LR children met DSM-5 criteria for ASD and none had a community clinical ASD diagnosis.

### Anxiety

The Spence Children’s Anxiety Scale Parent-Report (SCAS-P; Nauta et al. 2004) was used to measure anxiety symptoms at the 7-year visit. The scale measures anxiety within 6 domains, including separation anxiety, Obsessive Compulsive Disorder (OCD), panic/agoraphobia, social anxiety, physical injury fears, generalised anxiety, and a total anxiety score. The parent version consists of 39 items and asks respondents to report how frequently their child exhibits a range of anxiety-related behaviours (e.g. ‘My child worries that something bad will happen to him/her’). Responses are recorded on a 4-point Likert scale (never, sometimes, often and always). Total scores range from 0 to 112 and higher scores indicate more severe anxiety. The measure had excellent internal consistency in our sample, *α* = 0.92.

### Emotional Spatial Cueing Task

A modified version of the spatial cueing task (Posner et al. 1980) was used to measure attentional bias. The task was adapted to include emotional stimuli and has been previously used to measure both attentional engagement to and delayed disengagement from threatening stimuli in anxiety (e.g. Yiend and Mathews 2001).

### Stimuli

Sixty digitised colour photographs were selected from the International Affective Picture System database (IAPS; Lang et al. 2008) and were chosen because they had been used (or had similar content to those used) in previous studies of emotional picture processing in TD children (Hajcak and Dennis 2009; McManis et al. 2001). Of these, 20 were classified as threatening, 20 as neutral and 20 as positive^4^ based on ratings of affective valance and emotional arousal previously made by adult participants. A subset of these images were also rated by children aged 7–11 years (Lang et al. 2008). Threatening images included pictures of animals (e.g. snakes, spiders) and unpleasant scenes (e.g. injections) but none relied on human facial expressions to induce threat. Positive and neutral images were matched as closely as possible in content, colour, orientation, level of detail and brightness, through visual inspection.

Threatening images (*M* = 3.36, *SD* = 0.64) were rated by the IAPS sample (Lang et al. 2008) as less pleasant than neutral (*M* = 5.04, *SD* = 0.33) or positive (*M* = 7.44, *SD* = 0.50) ones and both threatening (*M* = 6.07, *SD* = 0.70) and positive (*M* = 5.44, *SD* = 0.80) images were rated as more emotionally arousing than neutral images (*M* = 2.78, *SD* = 0.50). Each picture subtended 4 by 3 inches and was presented either to the left or to the right of the fixation cross (4 inches between the centre of the fixation cross and the centre of the image) on a grey background. The task was presented on a 15-inch colour monitor and was programmed using E-Prime version 2.0 (Psychology Software Tools Inc. 2012).

### Procedure

Participants were given 30 practice trials with neutral stimuli, followed by 240 experimental trials in 4 blocks of 60 trials each. All 60 images (20 threatening, 20 neutral, and 20 positive) were presented within each block with equal presentations on the right and left of the fixation cross. Each image was presented once in every block, with both the order and assignment to congruent or incongruent trial randomised within each block.

Each trial began with a fixation cross at the centre of two empty rectangles (4 by 3 inches) for durations of 875–1275 ms. In order to minimise eye movements, participants were instructed to keep their eyes on the fixation cross throughout the task. Subsequently, an image (threatening, neutral or positive) appeared in either the right or the left rectangle for 500 ms. The image was then removed and replaced by a target (a star) at the centre of one of the rectangles and remained on screen until the end of the trial. In 70% of trials, the target appeared in the location of the image cue (congruent) and in 30% of trials the target was in the opposite location (incongruent). The uneven distribution of congruent and incongruent trials was done in order to facilitate covert orienting of attention in response to cueing. When a greater portion of trials are congruent, participants are more likely to covertly shift attention to the cued location because it is an accurate predictor of the target location most of the time, resulting in faster RTs on congruent trials and slower RTs on incongruent trials (Posner et al. 1980). Since enhanced attending is expected towards congruent trials, the slower RTs on incongruent trials are indicative of attentional disengagement.

Participants were asked to locate the target by pressing one of two buttons to indicate right or left. A new trial began once participants had made a response or after 3000 ms. The reaction time (RT) to detect the target was measured as the time, in milliseconds (ms), from target onset to button press. Feedback was given after each trial, indicating whether the response was correct, incorrect or if participants were too slow to respond. Mean RTs for each stimulus type (threatening, neutral and positive) in both congruent and incongruent trials were used in analyses.

### Statistical Analyses

#### Demographic Characteristics and Anxiety Symptoms

All data reduction and statistical analyses were carried out in SPSS version 20.0 (IBM Corp. 2011). Multivariate ANOVA and Chi square were used to compare groups on demographic characteristics. Anxiety symptoms were compared across the three groups and, as sex differences in anxiety are widely reported (McLean et al. 2011), we tested for sex differences in anxiety symptoms within our sample. To examine group and sex differences on anxiety symptoms, a 3 (group: HR-ASD, HR-non ASD, LR) x 2 (sex: male, female) ANOVA was run on the SCAS-P total score. Planned comparisons between each pair of groups were used where significant differences emerged, with Bonferonni correction applied for multiple testing. If a significant group x sex interaction emerged, follow up independent samples t-tests were run within each group to examine sex differences on anxiety scores, with Bonferonni correction applied for family-wise error related to multiple testing.

### Group Differences in Threat Bias

To examine group differences in threat bias, 6 indices of attentional engagement and disengagement were computed. Attentional engagement indices were computed by calculating the difference in mean RTs for non-threatening and threatening *congruent* trials. Three engagement indices were computed, including threat compared with neutral (“threat-neutral engage”), threat compared with positive (“threat-positive engage”) and positive compared with neutral (“positive-neutral engage”). Attentional disengagement was computed by calculating difference in mean RTs for threatening and non-threatening *incongruent* trials. Again three disengagement indices were computed comparing threatening with neutral (“threat-neutral disengage”), threatening with positive (“threat-positive disengage”) and positive with neutral (“positive-neutral disengage”).

Group differences in these 6 indices were compared between the 3 groups (HR-ASD, HR-non ASD, LR) using a MANOVA. Where significant group differences emerged, planned comparisons were carried out between each pair of groups, with Bonferonni correction applied for multiple testing. Furthermore, if group differences were detected on a particular bias index, follow-up tests were conducted to ensure that the bias score significantly differed from 0. To do this, one-sample t-tests were run on the selected bias score within each group, with Bonferonni correction applied for multiple testing.

Given that significant group differences emerged in FSIQ and there were sex differences in anxiety symptoms (see results), we repeated these analyses and co-varied for FSIQ and sex, to ensure that these factors did not alter the pattern of findings. Miller and Chapman (2001) suggest caution when using ANCOVA to control for group differences in measures such as IQ. Therefore, the results from these analyses are not included in the main text but are presented in the Supplementary Materials.

### Association Between Threat Bias and Anxiety

The association between threat bias and anxiety was examined in two steps. First-order Pearson correlations were run between each of the threat engagement and disengagement indices (threat-neutral engage, threat-positive engage, threat-neutral disengage and threat-positive disengage), SCAS-P total score, SRS t-score, and WASI FSIQ, with Bonferonni adjusted *p* values used to account for multiple analyses.

Because a significant association emerged between threat-positive engage and SCAS-P (see results), a follow-up linear regression was performed to assess the contribution of this attentional index to anxiety severity, co-varying for ASD severity and sex. As FSIQ was not significantly associated with SCAS-P total score or the threat-positive engagement index, it was not included the regression analysis. Cohen’s *d, η* ^*2*^ and *r* ^*2*^ were used to indicate the effect size (Cohen 1973).

## Results

### ASD Outcomes and Group Characteristics at 6–8 Years

Of the 42 HR children tested, 15 met DSM-5 criteria (American Psychological Association 2013) for ASD (HR-ASD), while 27 did not meet criteria (HR-non ASD) and none of the LR children exhibited evidence of ASD. Three children who had met diagnostic criteria for ASD at age 36 months no longer met criteria at age 7-years, therefore these participants were removed from further analyses, leaving a HR-non ASD group of *n* = 24. Table 1 provides demographic characteristics for the HR-ASD, HR-non ASD and LR groups. There were no differences among groups in age (*F*(2, 71) = 1.16, *p* = .31, *η* ^*2*^=0.03) or sex ratio (*X* ^*2*^ *(2)* = 3.48, *p* = .18). As expected, there were significant differences in SRS t-scores, *F*(2, 64) = 26.59, *p* < .001, *η* ^*2*^=0.45, where the HR-ASD group scored significantly higher than both the HR-non ASD (*p* < .001, *d* = 1.66) and LR (*p* < .001, *d* = 1.77) groups. The groups differed significantly on FSIQ, *F*(2, 70) = 3.25, *p* = .05, *η* ^*2*^=0.09, where the HR-non ASD group’s performance was significantly lower than the LR group’s (*p* = .05, *d* = 0.75), but there were no significant differences on either of the individual IQ subscales.

**Table 1:** Demographic characteristics, parent- and self-reported total anxiety scores and cognitive functioning for the HR-ASD, HR-non ASD and LR groups

Measure (SD)	HR-ASD (N = 15)	HR-non ASD (N = 24)	LR (N = 37)
Age (months)	89.13 (6.53)	91.42 (6.28)	89.26 (4.86)
Sex (male:female)	7:8	5:19	15:22
ADI—Social	13.14 (4.69)^a^	4.04 (5.48)^b^	N/A
ADI—communication	10.43 (4.59)^a^	4.25 (4.67)^b^	N/A
ADI—RRB	3.57 (1.74)^a^	0.58 (1.41)^b^	N/A
ADOS—CSS total	6.33 (2.92)^a^	2.46 (1.41)	1.70 (1.19)^b^
ADOS CSS SA	6.60 (2.59)^a^	2.96 (1.60)	2.18 (1.70)^b^
ADOS CSS RRB	6.13 (2.70)^a^	3.04 (2.84)^a^	1.12 (0.70)^b^
SCAS-P total score	26.20 (20.86)^a^	17.91 (8.55) (*N* = 23)	12.22 (7.27)^b^
SRS t-score	74.85 (22.77)^a^ (*N* = 13)	52.37 (11.74)^b^ (*N* = 19)	45.49 (5.82)^b^ (*N* = 35)
WASI full-scale IQ	109.79 (21.36) (*N* = 14)	107.96 (12.76)^a^	117.06 (11.61)^b^ (*N* = 35)
WASI verbal IQ	110.14 (25.87) (*N* = 14)	110.83 (14.94)	119.77 (13.93) (*N* = 35)
WASI performance IQ	109.57 (18.26) (*N* = 14)	102.71 (9.97)	110.34 (12.05) (*N* = 35)

Group sizes are smaller for some variables due to missing data. Groups denoted with different subscript letters (a, b, c) differed significantly with Bonferonni correction applied (p < .05). HR/LR indicates high-risk or low-risk group

Group differences in ADOS, ADI-R scores and SCAS-P subscales are reported in full in Shephard et al. (2016)

*ASD* autism spectrum disorder, *SD* standard deviation, *ADI-R* autism diagnostic interview – revised, *RRB* restricted repetitive behaviour, *ADOS* autism diagnostic observation schedule, *CSS* calibrated severity score, *SA* social affect, *SCAS-P* Spence children’s anxiety scale parent-report, *SRS* social responsiveness scale, *WASI* Wechsler abbreviated scale of intelligence

### Prevalence of Anxiety

Parent-report of anxiety symptoms, SCAS-P total score, revealed significant differences among groups, *F*(2, 68) = 9.87, *p* < .001, *η* ^*2*^=0.23. The HR-ASD group had substantially higher total SCAS-P scores than the LR group (*p* < .001, *d* = 0.89). The HR-non ASD group did not differ from the HR-ASD (*p* = .27, *d* = 0.52) or the LR (*p* = .08, *d* = 0.72) groups. There were significant sex differences in total anxiety levels (*F*(1, 85) = 11.08, *p* = .001, *ŋ* ^*2*^=0.14), where females (*M* = 18.50, *SD* = 13.96) had higher anxiety than males (*M* = 13.65, *SD* = 8.55), *d* = 0.42. There was also a significant group by sex interaction on the total anxiety score (*F*(2, 68) = 10.64, *p* < .001, *ŋ* ^*2*^=0.24) and to follow up on this interaction, independent samples t-tests were run within each group to examine sex differences on total anxiety. Bonferonni correction was applied to the *p*-value to account for family wise error related to multiple testing (0.05/6 = 0.008). The only significant difference emerged in the HR-ASD group, where females (*M* = 38.88, *SD* = 21.50) had significantly higher anxiety levels than males (*M* = 11.71, *SD* = 4.11), *t*(13)=-3.28, *p* = .001, *d* = 1.76, but there were no sex differences in the LR or HR-non ASD groups.

### Emotional Spatial Cueing Task

#### Preparation of RT Data

RTs on trials with incorrect responses or ones where the participant did not make a response were removed from further analysis. This resulted in removal of 4.41% of RT data from the HR-ASD group, 1.17% from the HR-non ASD group, and 3.48% from the LR group. Additionally, trials with RTs below 100 ms, which are indicative of automatic responding (Whelan 2008), and trials with RTs that were 3SD above the participant’s group mean were removed. This resulted in removal of a further 1.53% of RT data from the HR-ASD group, 2.87% from the HR-non ASD group, and 2.60% from the LR group. One participant from the HR-ASD group and 2 from the LR group had fewer than 50% valid trials in multiple conditions after removal of incorrect data and outliers, and were removed from further analyses. Additionally, 1 LR participant had unusually long RTs (+ 3SD compared to group RT) on multiple conditions and was also removed from further analyses. Two HR children were unable to complete the task due to having limited language and not being able to follow task instructions. A further 4 HR and 5 LR participants did not complete the task due to time constraints on the day of testing. This resulted in 35 HR (11 HR-ASD and 21 HR-non ASD) and 29 LR having useable RT data for analysis.

### Group Differences in Threat Bias

**Fig. 1:**
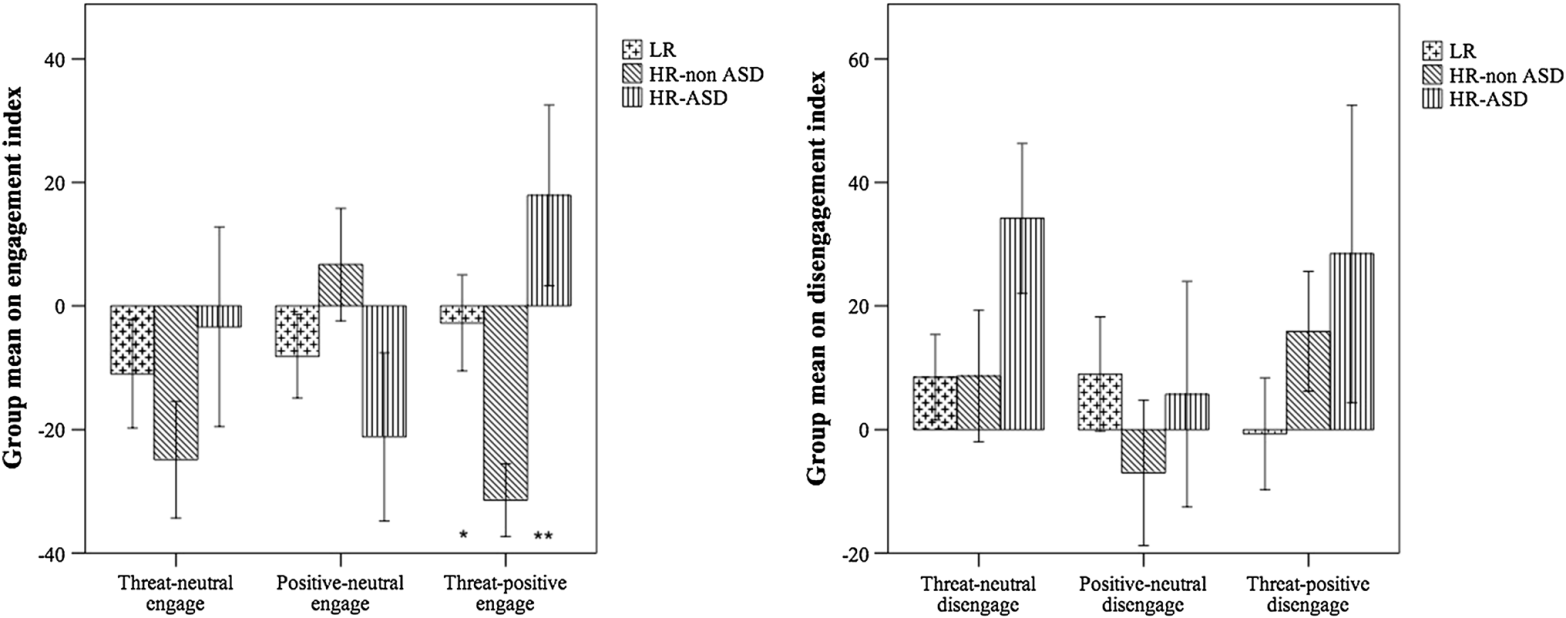
Threat engagement (difference in non-threatening and threatening congruent trials) and disengagement (difference in threatening and non-threatening incongruent trials) indices in the HR-ASD, HR-non ASD and LR groups with significant differences denoted with asterisks (*p < .05, **p < .01)

Figure 1 provides a summary of the engagement and disengagement index scores for each group. The MANOVA comparing the 6 attentional engagement and disengagement indices between the three groups revealed only one significant difference, in the threat-positive engagement index, *F*(2, 58) = 6.54, *p* = .003, *η* ^*2*^=0.18. Follow-up planned pairwise contrasts for the threat-positive engagement index revealed that the HR-non ASD group took significantly longer to engage with threatening stimuli (compared to positive stimuli) than both the HR-ASD (*p* = .003, *d* = 1.25) and the LR (*p* = .04, *d* = 0.82) groups.

**Fig. 2:**
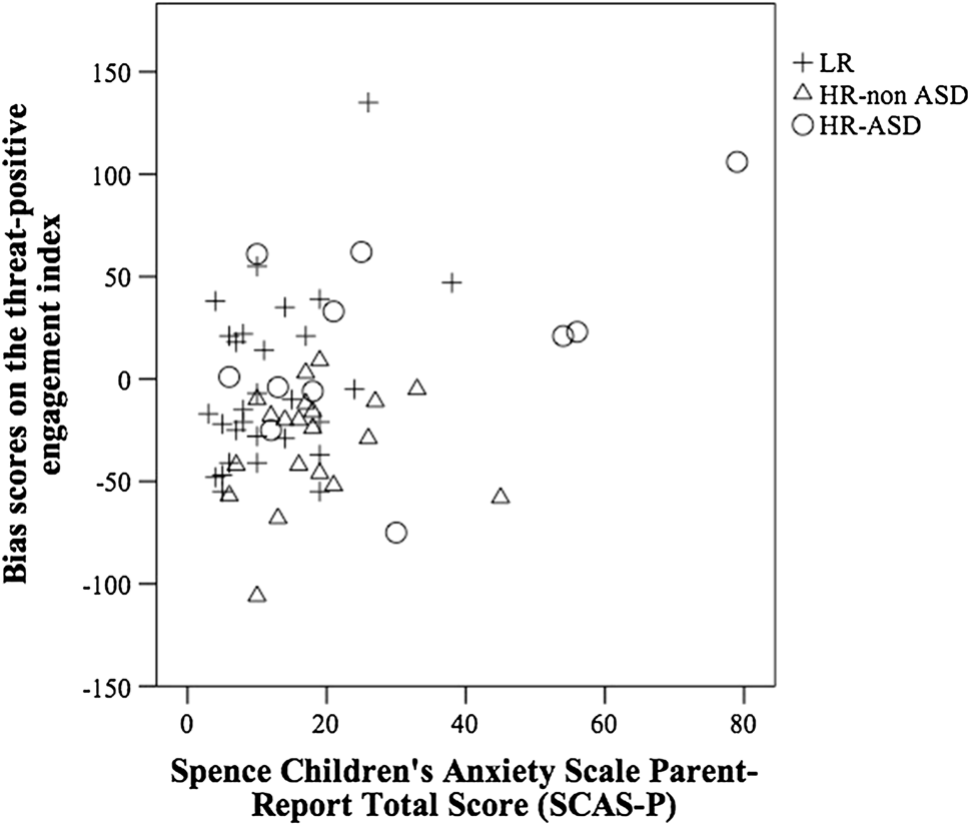
Association between the threat-positive engagement index and SCAS-P total score, with data points marked by group (HR-ASD, HR-non ASD and LR)

Follow-up, one-sample t-tests were run on the threat-positive engagement index within each group to confirm that this bias score was significantly different from 0. Threat-positive engagement was significantly different from 0 in the HR-non ASD group (*t*(20)=−5.32, *p* < .001), but not in the HR-ASD (*t*(10) = 1.22, *p* = .13) or the LR (*t*(20)=−0.35, *p* = .73) groups.

### Association Between Threat Bias and Anxiety Symptoms

There was a significant association between SCAS-P total score and the threat-positive engagement index, *r*(60) = 0.35, *p* = .01, *r* ^*2*^ = 0.12, but not any of the other attention indices (see Table 2). There was also a significant association between SCAS-P total score and SRS t-score, *r*(60) = 0.60, *p* = .01, *r* ^*2*^ = 0.36. Since FSIQ was not associated with SCAS-P total score or any of the threat bias indices, it was dropped from further analyses. Figure 2 shows the association between SCAS-P total score and the threatpositive engagement index in the HR-ASD, HR-non ASD and LR groups.

**Table 2:** First-order Pearson correlation coefficients (r) showing the association between each threat bias index, SCAS-P total score, SRS-2 and WASI-II FSQI

	SCAS-P	SRS-2	WASI-II FSIQ
Threat-neutral engage	0.19	0.00	0.00
Threat-positive engage	0.35*	0.21	− 0.07
Threat-neutral disengage	0.16	0.27	− 0.10
Threat-positive disengage	0.10	0.22	− 0.24
SCAS-P	1		
SRS t-score	0.60*	1	
WASI-II FSIQ	− 0.16	− 0.29	1

Associations denoted with an asterisk (*) were significant, with Bonferonni correction applied (*p* = .05/7 = 0.007). SCAS-P abbreviates the Spence Children’s Anxiety Scale-Parent Report; SRS-2 Social Responsiveness Scale; WASI-II FSIQ Wechsler Abbreviated Scales of Intelligence, 2nd Edition Full Scale IQ.

Further analyses were conducted to examine association between anxiety and threat bias, taking into account the contributions of ASD severity and sex. Linear regression was run with SCAS-P total score as the dependent variable, and the threat-positive engagement index as the independent variable, co-varying for SRS t-score and sex. The overall model accounted for a significant proportion of variance in anxiety symptoms, *F*(3, 49) = 20.61, *p* < .001, *r* ^*2*^ = *0*.56. Both the threat-positive engagement (*β*= 0.25, *t*(49) = 2.59, *p* = .01) and SRS t-score (*β*= 0.61, *t*(49) = 6.19, *p* < .001) were significantly associated with SCAS-P total score. Sex (*β*= 0.17, *t*(49) = 1.76, *p* = .08) had a trend-level association with SCAS-P total score.

## Discussion

The present study is the first to examine the association between attentional bias to threat, anxiety and ASD symptoms within the context of a high-risk for ASD sibling design. Attentional bias was enhanced in the HR-non ASD group, who exhibited longer latencies to detect threatening (compared with positive) stimuli than both the HR-ASD and LR groups. Engagement with threatening stimuli was significantly associated with anxiety symptoms, even after taking ASD severity and sex into account. On the contrary, while the HR-ASD group had elevated anxiety, they did not show evidence of threat bias. These findings suggest that the cognitive mechanisms associated with anxiety in non-ASD populations also relate to anxiety in non-affected siblings of children with ASD, but may not be present in those that have ASD.

### Attentional Bias to Threat in Children at High-Risk for ASD

The emotional spatial cueing task allowed exploration of multiple attentional systems (both attentional orienting and disengagement). We predicted that the HR-ASD group would exhibit delayed disengagement from threatening stimuli and that this would be associated with anxiety severity. However, several unexpected findings emerged. Firstly, in spite of having heightened anxiety, the HR-ASD group did not manifest delayed disengagement from or enhanced orienting towards threatening stimuli. On the other hand, the HR-non ASD group had significantly longer latencies when engaging with threatening, compared with positive, stimuli than both the HR-ASD and LR groups. Findings remained unchanged when sex and IQ were co-varied (see supplementary materials).

While the direction of bias observed in the HR-non ASD group is unexpected, numerous studies report prolonged latencies to engage with threatening stimuli and suggest this to be indicative of bias away from threat (e.g. Koster et al. 2006). Typically, such an attentional pattern is observed when stimuli are presented for long durations and there is sufficient time for conscious processing to occur (Koster et al. 2005; Mogg et al. 1997), but the time course of attentional processing in anxious children is less conclusive than in adults (Waters et al. 2010). However, multiple studies with both anxious adults and children report attentional avoidance when stimuli are presented for 500 ms, as they were in the experimental task used in this study (Koster et al. 2006; Waters and Kershaw 2015; Waters et al. 2012). Bar-Haim et al. (2007) suggest that individuals typically begin to process images consciously at approximately 500 ms and inconsistencies in previous studies could be largely due to methodological differences, such as use of colour vs. grey scale images and differential onset of target stimulus (Koster et al. 2006).

It is also important to note that attentional bias in the HR-non ASD group was observed when comparing threatening images with positive, rather than neutral, images. Given the evidence of atypical fear processing in individuals with ASD, we propose that the neutral images may have presented a certain level of ambiguity and more highly positive images were needed to offset the impact of the threatening stimuli. Research on fear conditioning in ASD suggests that individuals with the condition may have difficulty extinguishing previously learned fear associations (Top et al. 2016). This suggests that they have difficulty distinguishing between threat and safety cues and inhibiting fear responses when they are no longer relevant (Top et al. 2016; Waters et al. 2015). Furthermore, children with ASD are reported to have atypical fears and phobias, frequently of commonplace objects (Kerns et al. 2014; Evans et al. 2005). There is presently a scarcity of studies that explores fear processing in siblings of children with ASD. The threatening stimuli used in this study generally presented evolutionarily-relevant threats (e.g. snakes, spiders) or scenes depicting physical threat (e.g. injections, car crashes). Preschool children, as young as 3 years, exhibit enhanced attentional bias for evolutionary threat (Lobue and Deloache 2008). Our findings suggest that such threat stimuli are equally salient among unaffected siblings of children with ASD. However, future studies assessing threat bias in children with ASD or their siblings would benefit from asking them to rate the valance of the images.

### Threat Bias, Anxiety Symptoms and ASD Severity

A further aim of the present study was to examine the association between anxiety, threat bias and ASD severity. In addition to observing increased bias away from threat in the HR-non ASD group, we also found that anxiety was significantly associated with both threat bias and ASD severity. The association between heightened anxiety and ASD severity is unsurprising, as multiple studies report such an association among individuals with ASD (Sukhodolsky et al. 2008; Hallett et al. 2013a) and anxiety was most highly elevated in the HR-ASD group. The association between anxiety and the threat-positive engagement index remained significant even when taking into account ASD severity and sex. This implies that the increased threat bias observed in the HR-non ASD group is not merely a by-product of having symptoms of ASD, but is uniquely associated with anxiety. While the difference was not significant, the HR-non ASD group did have higher mean scores on the anxiety measure than LR controls at trend-level, which may have reached significance with a larger sample size. This evidence suggests that anxiety functions similarly in non-affected siblings of children with ASD as it does in non-ASD populations. Furthermore, longitudinal studies in non-ASD populations suggest that increased attentional bias to threat in childhood is a risk factor for the development of anxiety related difficulties in adolescence (Perez-Edgar et al. 2010). Therefore, the elevated threat bias observed in the HR-non ASD group could indicate risk for the development of more severe anxiety in later development.

The HR-ASD group, on the other hand, had markedly higher anxiety levels compared to LR across multiple domains but did not exhibit attentional bias to threat. While it is possible that the modest size of the HR-ASD group (*n* = 11) meant that there was insufficient power to detect a significant difference, the HR-non ASD group did have significantly higher threat bias than HR-ASD group, with a large effect size (*d* = 1.25; Cohen 1973). Multiple studies have reported elevated rates of anxiety in individuals with ASD, but found no evidence of an association between anxiety symptoms and bias to socially threatening stimuli (Hollocks et al. 2013; May et al. 2015). In this study, we failed to observe an association between anxiety and bias to non-social threat. Given these findings, it is possible that anxiety among ASD populations is not characterised by biased attentional allocation to threat, but that different mechanisms are involved. For example, increased anxiety within ASD may be more attributable to worries about uncertainty (e.g. Wigham et al. 2014), fear of unwanted change and reduced ability to cope with distress, rather than biased attentional allocation to threat (Hollocks et al. 2013). Thus, it is possible that the stressors associated with anxiety in ASD cannot easily be portrayed using visual stimuli.

Finally, accurately assessing anxiety symptoms among individuals with ASD is highly challenging (Wood and Gadow 2010). One of the most prominent factors is the discrepancy observed in self- and caregiver- report of anxiety symptoms and reduced sensitivity of current measures in ASD-populations (Mazefsky et al. 2014). There are also challenges in disentangling symptoms of anxiety and the core features of ASD (Kerns and Kendall 2012) and obtaining accurate accounts of anxiety symptoms among individuals with ASD and reduced intellectual ability (Sukhodolsky et al. 2008). One of the most important criticisms of current measures is that they do not accurately capture the construct of anxiety within ASD, particularly as there are features of anxiety, such as intolerance of uncertainty, heightened sensory sensitivity and atypical fears, that are prevalent among individuals with ASD but not commonly observed in non-ASD individuals with anxiety (Rodgers et al. 2016; Kerns et al. 2014). To our knowledge, the reliability of parent-reported anxiety symptoms in non-affected siblings of children with ASD has not yet been examined. The association between parent-reported anxiety and threat bias reported in this study suggests that parents are able to reliably report on anxiety symptoms in their children who do not have ASD. It is also possible that parents of children in the HR-ASD group may have overestimated anxiety severity.

### Strengths, Limitations and Implications for Future Research

The present study is the first to explore symptoms of anxiety and attentional bias to threat in children with increased familial risk for ASD. The findings have implications for both research and clinical practice. Our findings suggest that in non-affected siblings, the cognitive correlates of anxiety are similar to those found in non-ASD populations. However, the HR-ASD group did not exhibit heightened bias to threat, in spite of having elevated anxiety by parent report. In line with previous research, this finding could suggest that the cognitive correlates of anxiety in children with ASD are different from those observed in anxious individuals without ASD. Further investigation is required to understand the neurocognitive mechanisms that underlie anxiety in ASD. This could have important implications for clinical practice, as existing therapies for anxiety may need to be modified to suit the specific needs of children with ASD, particularly as threat bias modification therapy is showing increasingly promising results in treating anxiety in children (Shechner et al. 2014).

One limitation of the present study was the small sample size, particularly within the HR-ASD group. Because of this, we were unable to examine associations between threat bias and anxiety independently for each group. We were also unable to explore these associations in relation to clinically diagnosed anxiety, only a dimensional measure of anxiety symptoms. Future research should examine whether the association between threat bias and anxiety is present in children who are at high-risk for ASD who meet diagnostic criteria for anxiety disorders. A further limitation is that the highly varied nature of the IAPS images meant that it was difficult to control the visual properties (e.g. luminance, spatial frequency and colour) of the stimuli used in the emotional spatial cueing task. However, to control for a possible mismatch in the visual properties of the stimuli, each image was presented once in every block, with both the order and assignment to trial type (congruent/incongruent) randomised to ensure that no one image was presented in a particular location or trial type, thus reducing the potential for particular images biasing participants’ attention.

It is also unusual that sex differences in anxiety were present mainly in the HR-ASD group and not the HR-non ASD or LR groups, particularly as sex differences in anxiety are highly prevalent from a young age (Mclean et al. 2011). It is possible that the modest size of the different groups meant we did not have statistical power to detect differences. However, within the HR-ASD group, sex differences were more highly pronounced, allowing the sex difference to reach significance.

Finally, there is a need for longitudinal studies to explore the development and trajectories of anxiety in ASD and non-ASD siblings. Such studies would help elucidate the cause of such high co-occurrence of the two conditions.

## Electronic supplementary material

Below is the link to the electronic supplementary material.

Supplementary material 1 (DOC 27 KB)

## Footnotes

1 5 DAWBA and 5 SCQ missing.

2 One infant was not born at full-term.

3 1 SCQ missing.

4 The following IAPS images were used: ***Threatening*** (1050, 1120, 1201, 1300, 1525, 1930, 1932, 3210, 6190, 9312, 6370, 9373, 9440, 9480, 9590, 9592, 9622, 9902, 9909, 9940), ***Neutral*** (2038, 2396, 2579, 5390, 5520, 5530, 5740, 7004, 7006, 7025, 7035, 7050, 7060, 7100, 7140, 7150, 7175, 7217, 7233, 7595) and ***Positive*** (1710, 1750, 1920, 1999, 2650, 5450, 5460, 5470, 5480, 5621, 5910, 7250, 7270, 7330, 7430, 8200, 8260, 8420, 8490, 8510).
