# Supplementary material for: Anxiety and Attentional Bias to Threat in Children at Increased Familial Risk for Autism Spectrum Disorder
Source: J Autism Dev Disord. 2017 Jan 23;47(12):3714–27. doi: 10.1007/s10803-016-3012-1 (PMC5676835; doi:10.1007/s10803-016-3012-1)
Supplement: Supplementary file 1 — Supplementary material 1 (DOC 27 KB) [file 10803_2016_3012_MOESM1_ESM.doc]

Anxiety and Attentional Bias to Threat in Children at Increased Familial Risk for Autism Spectrum Disorder

**Supplementary materials**

*Prevalence of anxiety, when co-varying for FSIQ*

To examine group and sex differences on anxiety symptoms, a 3 (group: HR-ASD, HR-non ASD, LR) x 2 (sex: male, female) ANCOVA was run on the SCAS-P total score, co-varying for IQ. Planned comparisons between each pair of groups were used where significant differences emerged, with Bonferonni correction applied for multiple testing. If a significant group x sex interaction emerged, follow up independent samples t-tests were run within each group to examine sex differences on anxiety scores, with Bonferonni correction applied for family-wise error related to multiple testing. As there were significant sex differences and a group x sex interaction on anxiety scores (see results), sex was also co-varied for in further analyses.

Parent-report of anxiety symptoms, SCAS-P total score, revealed significant differences among groups, *F*(2, 64)=8.45, *p*=.001, *η^2^*=.209. The HR-ASD group had substantially higher total SCAS-P scores than the LR group (*p*<.001, *d*=.89), whereas the HR-non ASD group did not differ from either the HR-ASD (*p*=.223, *d*=.52) or LR (*p*=.138, *d*=.72) groups.

There were significant sex differences in total anxiety levels *F*(1, 64)=9.67 *p*=.003, *d*=.42. Females (*M*=18.50, *SD=*13.96) had higher anxiety than males (*M=*13.65*, SD*=8.55). There was also a significant group x sex interaction on the total anxiety score *F*(2, 64)=8.47, *p*=.001, *η^2^*=.215. To follow up on this interaction, independent samples t-tests were run within each group to examine sex differences on total anxiety. Bonferonni correction was applied to the *p*-value to account for family wise error related to multiple testing (.05/6=.008). The only significant difference emerged in the HR-ASD group, where females (*M*=38.88*, SD*=21.50) had significantly higher anxiety levels than males (*M*=11.71*, SD*=4.11), *t*(13)=-3.28, *p*=.001, *d*=1.76, but there were no sex differences in the LR or HR-non ASD groups.

*Group differences in threat bias, co-varying for FSIQ and sex*

A 3 (Group: HR-ASD, HR-non ASD, LR) x 6 (Index) MANCOVA was run, co-varying for FSIQ and sex. Only one significant difference emerged, *F*(2, 56)=7.52, *p*=.001, *η^2^*=.212, on the threat-positive engagement index. Follow-up analyses revealed that the HR-non ASD group took significantly longer to engage with threatening stimuli (compared to positive stimuli) than both the HR-ASD (*p*=.002, *d*=1.25) and the LR (*p=*.015, *d*=.82) groups.
